# Electromagnetic deactivation spectroscopy of human coronavirus 229E

**DOI:** 10.1038/s41598-023-36030-6

**Published:** 2023-06-01

**Authors:** Hayden Banting, Ian Goode, Carla E. Gallardo Flores, Che C. Colpitts, Carlos E. Saavedra

**Affiliations:** 1 Electrical and Computer Engineering, Queen’s University, Kingston, K7L 3N6 Canada; 2 Biomedical and Molecular Sciences, Queen’s University, Kingston, K7L 3N6 Canada

**Keywords:** Microbiology, Engineering

## Abstract

An investigation of the deactivation of pathogens using electromagnetic waves in the microwave region of the spectrum is achieved using custom-built waveguide structures. The waveguides feature sub-wavelength gratings to allow the integration of an air cooling system without disturbing the internal propagating fields. The waveguides are tapered to accommodate an experimental sample internally with sufficient surrounding airflow. The proposed methodology allows for precise control over power densities due to the well-defined fundamental mode excited in each waveguide, in addition to temperature control of the sample due to microwave exposure over time. Human coronavirus (HCoV-229E) is investigated over the 0–40 GHz range, where a peak 3-log viral reduction is observed in the 15.0–19.5 GHz sub-band. We conclude HCoV-229E has an intrinsic resonance in this range, where nonthermal structure damage is optimal through the structure-resonant energy transfer effect.

## Introduction

The deactivation of pathogens using electromagnetic (EM) waves in the microwave band is attracting growing research interest^1–9^. The contactless nature of microwave deactivation is a feature that makes the method particularly useful in the context of the public health crises caused by the recent and ongoing SARS-CoV-2 pandemic. Microwaves can deactivate a virion in one of two ways: through thermal heating or through a process known as structure-resonant energy transfer (SRET). The latter is based on the idea that enveloped viruses with simple spherical geometries will resonate in the presence of an EM wave^2–5^. Maximizing the amplitude of the acoustic vibrations excited within a spherical virus is important to cause the greatest displacement and stress on the envelope structure, which may eventually cause it to rupture. Current modelling of acoustic dipolar-mode vibrations in spherical viruses predict the greatest stress applied from equal-intensity EM waves occurs in the microwave regime^2,4,10^, which is supported by a growing body of experimental evidence^2,3,5^. Deactivation of Influenza A (H3N2) virus has been demonstrated using low-power density microwaves, in which the virus membrane is ruptured through the SRET effect^2^. In that study, a viral solution saw a 3-log reduction of active virus after 15 minutes of microwave illumination from a horn antenna operating at 8.2 GHz. Application of the SRET effect is a promising nonthermal means to deactivate harmful pathogens with intrinsic resonances in the microwave regime due to the suggested low power densities required^2–4^.

Low-power non-heating microwave sterilization requires knowledge of the virion intrinsic natural resonance in order to most efficiently transfer as much of the limited energy available. Experimentally studying the microwave absorption spectroscopy of a virus is technically challenging, namely due to the sensitivity required to reasonably detect and distinguish a response attributed to the small-sized particles. Methods proposed have involved microwave transmission lines in which small volumes of solution are introduced to disturb the guided microwaves within the structure^2,3,5,8^. The sensor is first measured with only the carrier fluid as a reference, and then followed by a measurement containing some concentration of virus. A relative comparison is then made to identify regimes where more microwave power is lost, indicating absorption by the virus. This methodology has been used to identify microwave absorption resonances of SARS-CoV-2^3^, Influenza A (H3N2)^2^ and white spot syndrome virus^8^.

In this report, we present a novel temperature-controlled methodology to study electromagnetic interactions with pathogens. Human coronavirus HCoV-229E (229E) is selected to be used as a surrogate biosafety model for more highly pathogenic coronaviruses. It’s spherical geometry and spike protein arrangement is representative of many enveloped viruses. Our methodology is demonstrated by studying the SRET-based deactivation of 229E covering 0.8–40 GHz and identifying an intrinsic resonance within the 15.0–19.5 GHz regime. Within this regime, a 3-log reduction of active virus was observed after only 7.5 minutes of microwave exposure. Rectangular waveguides are used which are designed to accommodate a sample internally, exposing the sample to well-defined electric fields. This possesses the key advantage of having precise control over field intensity and power density exposed to the experimental sample. Sub-wavelength gratings are introduced into the waveguide walls to integrate an airflow cooling system without disturbing the propagating fields. During experiments, viral samples are continuously cooled to provide confidence that any deactivation observed is attributed to the SRET induced acoustic vibrations, rather than excess microwave heating of the carrier solution. Using this methodology, viruses can be studied under different power density and time criteria, such that optimal frequency regimes and expected degree of virus deactivation can be determined. This information is critical for the development of new microwave-based technologies for transmission control, sterilization, and clinical treatments.

## System design

### Design of air-cooled waveguides

**Figure 1:**
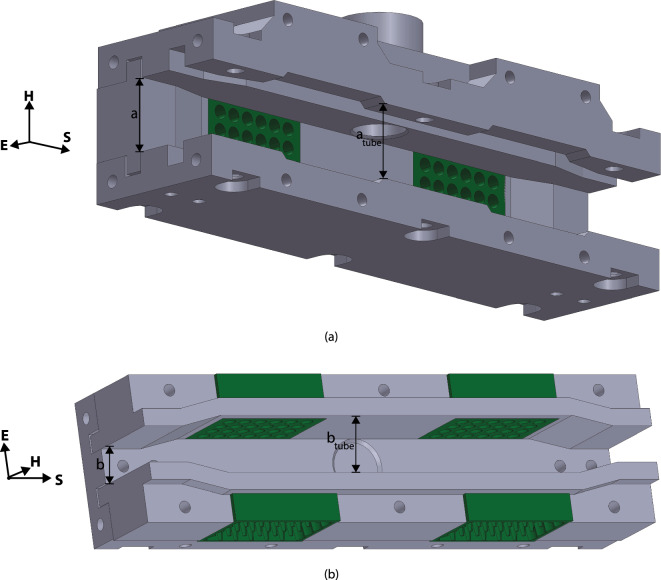
(**a**) Top and (**b**) Side view of a waveguide sensor showing the dimensions of the feed (*a* and *b*) and the dimensions of the waveguide around the test tube. Image generated from the CAD model of the waveguides designed in Dassault Systemes Solidworks (Version 2021).

To uniformly radiate the viral samples while controlling for temperature, a series of waveguides were designed to direct the radio frequency (RF) power toward the sample and also allow for air to flow past the sample to maintain temperature. These waveguides were designed to radiate samples across as wide a bandwidth as possible while operating in the fundamental mode, using commercial waveguide launchers when possible. This dictated the use of many waveguide bands to cover a large swath of spectrum, as shown in Table 1. Table 1 shows the dimensions of the feed waveguide (*a* and *b*) as well as the dimensions of the waveguide where the tube was located ($a_{tube}$ and $b_{tube}$). An example of these dimensions is shown in Fig. 1. This image was generated from the CAD (Computer Aided Design) model used to design the waveguides. All mechanical CAD work was completed in Dassault Systems Solidworks 2021.

Coaxial to waveguide launchers were used in all cases, except at the lower band (0.8–1.8 GHz) for a printed circuit board (PCB) waveguide probe feed in a reduced height waveguide was designed. All test-tubes were filled with $600~\mu l$ of fluid and were located in the center of the waveguide, with the fluid positioned at the center of the waveguide cross-section to maximize the exposure of the sample to the electromagnetic field. At higher frequencies, this required expanding $a_{tube}$ and $b_{tube}$ relative to *a* and *b* to fit the viral sample fully in the waveguide and allow space between the test tube and the walls of the waveguide for cooling.

**Table 1:** Dimensions of Air Cooled Waveguides.

WR Designation	Spectral range (GHz)	*a* (mm)	*b* (mm)	$a_{tube}$ (mm)	$b_{tube}$ (mm)
Reduced WR975	0.8–1.8	309.1	50.8	309.1	50.8
WR284	2.1–4.4	72.1	34.1	72.1	34.1
WR187	3.4–6.4	47.6	22.2	47.6	22.2
WR137	5.9–8.2	34.9	16.0	34.9	16.0
WR90	8.2–12.4	22.9	10.2	22.9	14.0
WR62	12.4–19.5	15.8	7.9	15.8	14.0
WR42	20.0–26.5	10.7	4.3	16.5	14.0
WR28	26.5–40.0	7.1	3.6	16.5	14.0

**Figure 2:**
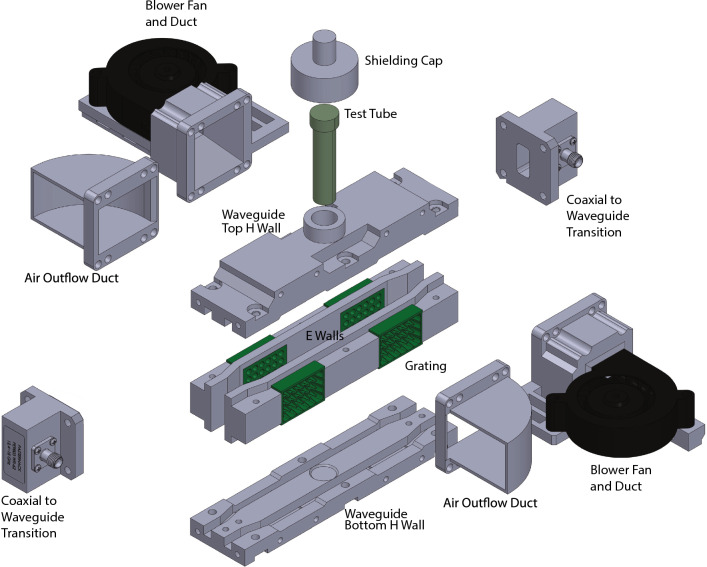
General assembly of waveguide sensor. Image generated from the CAD model of the waveguides designed in Dassault Systemes Solidworks (Version 2021). Models for the WR90 launchers were provided for free from ^11^.

The test tube samples are cooled by forcing air through the waveguide and past the sample tube. This was accomplished using blower fans as shown in Fig. 2 that forced air through grates in the side of the waveguide, past the sample, and exiting through an exhaust duct. These grates were metallized and designed to have holes large enough to let air flow through, yet small enough relative to the signal wavelength to contain the bulk of the RF energy inside the waveguide.

**Figure 3:**
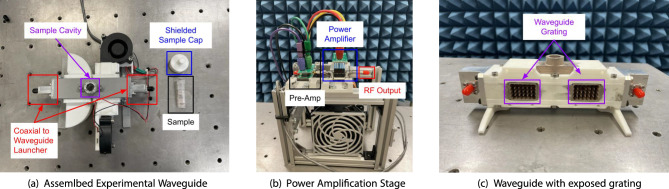
Images of assembled waveguide components and power amplifier used to complete the viral deactivation experiments, each of the waveguides was designed and printed by the group, and the amplifier was assembled by the group using off the shelf components.

**Figure 4:**
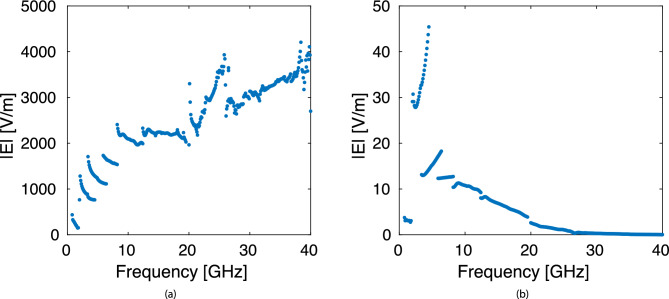
Simulated field intensities at the center of the test tube when the test tube was filled with (**a**) air or (**b**) reduced-serum media.

The waveguides were 3D printed using fused deposition modeling (FDM) from polylactic acid (PLA) and metallized using aluminum tape. This technique is convenient for rapid prototyping of waveguide parts with performance on par with all-metal components ^12,13^. This method was used to assemble each of the waveguide bands shown in Table 1 except for the lowest band. Below 1.8 GHz, the waveguide was implemented by folding sheet metal to form the walls and a scaffolding frame was 3D printed to hold the metal sheets together. As noted earlier, this waveguide was fed using a PCB probe. Sample images of the assembled waveguides are shown in Fig. 3. The waveguide structures were simulated using a full-wave finite-element solver (ANSYS HFSS) to determine the field intensities at the center of the viral sample tube. The input power was set to 2 Watts and simulations were performed for the case of an empty tube filled with air and the case of a tube filled with the dielectric model for the viral carrier solution. The simulated field intensities are shown in Fig. 4. The field intensities for the fluid filled test tube compared to the air filled test-tube (especially at higher frequencies) is due to the increasing $\epsilon _r$ of the carrier solution with frequency compared to air.

## Results

### Dielectric properties of reduced-serum medium

**Figure 5:**
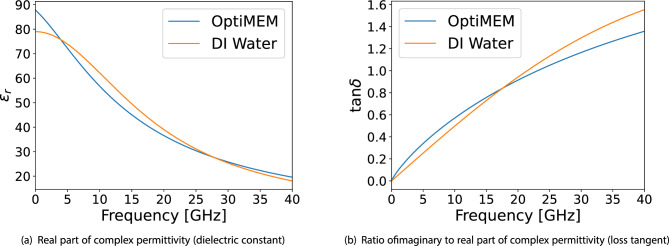
Dielectric model of reduced-serum medium compared to deionized (DI) water.

The complex permittivity provides a full time-harmonic EM description of a medium. The complex permittivity of the reduced-serum medium (OptiMEM) without any virus is characterized using the open-ended coaxial probe method. A frequency-dependent model is generated using a previously developed methodology^14^ and is shown in Fig. 5 along with deionized (DI) water for reference. The high dielectric constant of the reduced-serum medium can cause significant reflections within the air-filled waveguide, and if not unaccounted for in design, the input match of experimental waveguides could be degraded severely. For this reason, the permittivity model is integrated within the EM simulation tools in order to help design, tune, and evaluate waveguide structures such that high RF performance is achieved in the experimental cases which contain samples. Since the viral concentration in the samples is low, it was assumed that there would be minimal impact to the dielectric properties of the reduced-serum media, regardless of the presence of the virus.

### Microwave heating of reduced-serum medium

Microwave power absorbed by experimental samples causes both heating and disruptions to the virus structure. Without a cooling system to remove excess heat, virus temperature could reach sufficient criteria such that heat-related reduction would be observed. Heating of reduced-serum medium due to microwave exposure during experimental procedures is characterized to verify the integrated cooling system is able to sufficiently remove excess heat and ensure any observed viral deactivation would not be attributed to heat. Specifically, in all experiments the virus temperature must not exceed 44 $^\circ$C, as some degree of heat-related deactivation of HCoV-229E would be expected. Other coronaviruses are known to show inactivation when heated in the range of $44^\circ C-65^\circ C$ over comparable short time-scales (< 15 mins) to those used in this work^15^. Some variation of experimental sample heating is observed over the spectrum investigated in this study. Even though input power is constant for all experimental cases, the variation of waveguide dimensions vary the power density of guided waves, as well as alter the space surrounding the sample for air to flow. Additionally, the dielectric properties of the reduced-serum medium vary with frequency, namely the loss tangent, which influences the fraction of power absorbed by the media. For these reasons, sample heating characterization must be conducted for all cases individually in order to verify the sample is sufficiently cooled. The increase in temperature with respect to nominal room temperature (25 $^\circ$C) during deactivation experiments is summarized in Table 2. In all cases, amassing sample heating remains below 15 $^\circ$C, ensuring no experimental sample would exceed 40 $^\circ$C in all cases.

### Viral deactivation results

**Figure 6:**
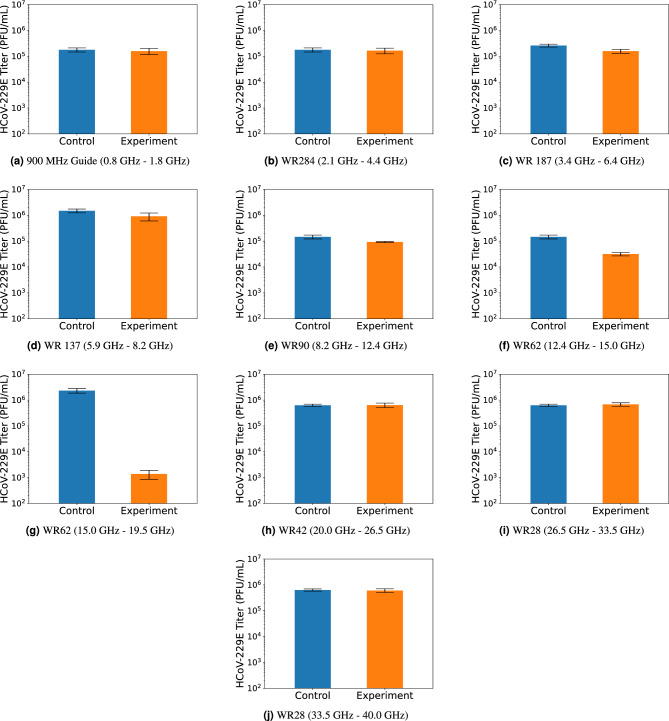
Virus Inactivation in Response to Microwave Exposure.

Deactivation of HCoV-229E is studied in subdivisions of the entire frequency spectrum up to 40 GHz. Multiple samples containing equal concentration of HCoV-229E are prepared for every sub-band, such that the experiments can be repeated several times to improve confidence in the results. The samples are divided into experimental and control groups, where only experimental samples are inserted into the waveguide structure and exposed to a range of frequencies. The details of the experimental sweep plan for each sub-band is provided in the methodology section. Plaque assay analysis is used to determine the active viral concentration after exposure to microwaves, which is compared to the control sample to establish the relative viral reduction. For all bands, the average viral reduction over all trials is shown in Fig. 6, each with an error bar representing the standard deviation of the experimental set. These results are summarized in Table 2. If the average reduction is less than tenfold when compared to the control, or the experimental and control sets have overlapping standard deviation intervals, “insignificant” is reported under the “Viral Reduction” column in Table 2 for that experiment. For all bands, the viral reduction observed is shown in Fig. 6 and is summarized in Table 2. Viral reduction must be statistically significant as well as greater than tenfold, otherwise “insignificant” is reported for the respective band.

**Table 2:** Summary of Virus Inactivation Experimental Results.

WR Designation	Spectral range (GHz)	Sample Heating ($^\circ ~C$)	Viral Reduction (-)
Reduced WR975	0.8–1.8	0	insignificant
WR284	2.1–4.4	0	insignificant
WR187	3.4–6.4	5	insignificant
WR137	5.9–8.2	7	insignificant
WR90	8.2–12.4	15	insignificant
WR62	12.4–15.0	10	1-log
WR62	15.0–19.5	2	3-log
WR42	20.0–26.5	3	insignificant
WR28	26.5–33.5	3	insignificant
WR28	33.5–40.0	3	insignificant

## Discussion

The primary findings of this study reveal an intrinsic resonance of HCoV-229E located within the 15.0–19.5 GHz frequency regime. After 7.5 minutes of microwave exposure, a 3-log reduction is observed in active virus concentration within this band. This level of viral deactivation is comparable to that observed in other SRET studies, although is achieved over a shorter duration. Additionally, a 1-log reduction was observed in the adjacent band of 12.4– 15.0 GHz, indicating some sensitivity, but not as optimal and efficient near the resonance. Outside the 12.4–19.5 GHz range, no substantial and statistically significant reduction was observed. The greatest sample heating occurred in the 8.2– 12.4 GHz range, reaching 40 $^\circ$C during the experiments, although no significant reduction was observed. This result further supports that observed viral reduction in other bands is attributed to structure damage due to the SRET effect as opposed to heating.

For the purpose of viral deactivation spectroscopy, rectangular waveguides are advantageous when compared to other guided (microstrip lines, coplanar waveguide) and radiating (antenna) solutions. Firstly, the fields within rectangular waveguides have well-defined propagating modes. This makes determining the electric fields and power density within the waveguide, even in the presence of an experimental sample, more precise. This is particularly useful in cases where the interest is to study a pathogen’s deactivation response to various field intensities or power densities. Additionally, rectangular waveguides can be tapered to provide additional space for convenient integration of an experimental sample, as was done in this work. This will alter the cross-section of the guide where the sample is located, but not the propagating modes, and so power densities and electric field intensities can still be precisely determined. Printed circuit board transmission lines, such as microstrip lines or coplanar waveguides, are more limited in terms of available space and ease of integrating an experimental sample. In these cases, experimental samples can only be practically placed above the signal-side conductor. However, doing so is extremely inefficient in terms of sample field exposure, as the fields are primarily contained within the substrate between the top-side signal and reverse-side ground conductors. Systems which radiate a virus sample using antennas share the same ease of sample integration as rectangular waveguides, however, they are not well suited for high power settings. While the antenna hardware can handle high power, radiating at these levels requires additional absorbing materials and RF shielding to be executed safely, and are subject to additional regulations and restrictions^16,17^. Conversely, rectangular waveguides can handle and self-contain high power levels, minimizing RF exposure risks to personnel involved conducting the experiments.

The development of microwave-based sterilization systems, clinical treatments, and other transmission control technologies fundamentally rely on knowledge of which frequencies to utilize and the degree of active viral reduction to expect over a given time. The viral deactivation spectroscopy methodology described in this article has been demonstrated to produce this information for HCoV-229E, and is also applicable to study other electromagnetic interactions with pathogens in the microwave regime. Additionally, the proposed methodology can be used to study the effects of incident power levels as well as exposure duration in relation to SRET virus reduction, which are known to have influence^2^ In general, exposing a virus sample to microwaves causes some degree of heating in addition to induced acoustic vibrations within the virions. However, the integrated temperature control of the experimental waveguides provides confidence that the observed viral reduction is attributed to structural damage from acoustic vibrations as opposed to excess heating. In terms of pathogen transmission control, heating can be undesirable. In sterilization settings, many materials may degrade or become damaged if heated for long durations or to relatively high temperatures. Low-power microwave technologies are promising for these settings, as significant active virus reduction is possible while maintaining safe levels of heating.

## Conclusion

Presented in this report is a novel methodology for studying functional virus inactivation in response to electromagnetic fields. The proposed methodology was demonstrated by studying 229E over the microwave spectrum and identifying regimes with clear and significant reduction of viral infectivity. As described within our “Discussion” section, this information can be used to develop and optimize transmission control technology targeting similar spherical viruses. Further studies beyond the scope of this work would be required to confirm precise mechanisms of virus structure damage in response to electromagnetic fields.

## Methods

### Waveguide fabrication and assembly

The air cooled waveguide was designed to be 3D printed from PLA and metallized using aluminum foil tape. This design methodology has been previously proven in ^12,13,18^ and allows for rapid prototyping of waveguide components. Each waveguide was printed in four sections, which allowed the aluminum tape to be applied to a flat surface to reduce wrinkling. The sections of the waveguide were slotted and screwed together. The waveguides each were fed by standard off-the-shelf waveguide launchers and were tapered to accommodate the size of the viral sample tube. To cool the samples, holes were cut into the sides of the waveguide and filled with a grating. This grating allowed air to pass through the waveguide, but contain the RF power inside the guide. For each waveguide these were printed as a separate piece and were metallized on the waveguide side with aluminum tape and on the outside with a conductive copper based paint (MG-Chemicals 843WB) to increase the isolate. Each waveguide had four of these gratings; two had axial fans to force air into the waveguide, and the other two acted as exit ports with ducts to direct the warm air away from the fan intake. Finally, each waveguide included a location for the sample tube. This was a hole in the side of the waveguide that fit snugly to the test-tube and was metallized and had a metallized shielding cap that together contained the RF energy in the waveguide.

### Microwave generation and amplification

A signal generator (Anritsu MG3694A) is used to generate microwave tones at desired frequencies. Power amplification stages are used to increase the power of the signal generator tones such that 2W of power is delivered to the experimental waveguide input. Multiple power application configurations were required to cover the large spectrum studied. In the 0.8–8.2 GHz range, five package amplifiers (Analog Devices HMC659LC5) are used: one in series and four in parallel, which are power combined to reach the target power level. In the 8.2–19.5 GHz and 20–40 GHz ranges, a single packaged amplifier (Mini-Circuits ZVE-3W-183+ and Qorvo QPA2640D, respectively), is used for power amplification. The power response of all amplification stages is characterized by sweeping the incident power from the signal generator and measuring the output power on a spectrum analyzer (Anritsu E4446A). This procedure is repeated at every frequency used in the experimental virus deactivation sweep plan. Thereafter, the power amplification characterization data is saved to memory of a digital-assist embedded system which interfaces with the signal generator. This system corrects intrinsic frequency-variations within each power amplification stage by adjusting the incident signal generator power such that the output power will be precisely 2W (33 dBm) at all frequencies.

### Virus deactivation experiments

The 0.8–40 GHz spectrum investigated is discretized into 10 sub-bands based on the supported frequency range of each waveguide designation used, which are summarized in Table 2. The experimental range of 0.8– 40 GHz was selected to include and surround those in^2,3^ where resonances for comparable viruses were observed. Multiple waveguides were used to cover this band so that each test could be carried out at the waveguide’s fundamental frequency. It was crucial to operate in the waveguide’s fundamental frequency so that the field max was in the center of each viral sample. Each sub-band uses an identical microwave sweep plan, consisting of 10 equally-spaced discrete tones within its respective band. The microwave generation and amplification stage produces each tone with incident power of 2W to the waveguide for 45 seconds, in ascending order, for a total sweep time of 7.5 minutes. The total sweep time was selected to be comparable to that in^2^ which achieved significant viral reduction was observed for. Equal-concentration live virus samples are prepared and split into experimental and control groups. Both groups, control and experimental, each contain three samples so that every experiment (sub-band) can be repeated three times to analyze repeatability and average virus reduction. All samples are stored in an ice bath for the duration of the experiments. Experimental samples are temporarily removed from the ice bath and inserted into the waveguide, which thereafter are exposed to propagating microwave fields according to the sweep plan described. Control samples receive no exposure to microwaves. For every sub-band, the experiment is repeated for three trials. Plaque assay analysis is used to determine the average reduction in active virus of experimental samples relative to the control samples.

### Reduced-serum medium heating characterization

Reduced-serum medium (OptiMEM) heating is characterized to determine the amount of virus heating during virus deactivation experiments. A sample is prepared containing equal volume of medium used in experimental trials. Firstly, the temperature of the sample is measured to determine ambient room temperature. Thereafter, the sample is inserted into the experimental set-up where the virus deactivation sweep plan is executed, exposing the sample to microwaves within the waveguides. Upon completion of the sweep plan, the sample temperature is immediately measured to characterize any heating due to microwave exposure. This procedure is repeated for every sub-band included in this study in order to verify that sample heating is sufficiently low and would not contribute to virus deactivation in all cases.

### Reduced-serum medium complex permittivity characterization

The open-ended coaxial probe method is used to measure the complex permittivity of the reduced-serum medium. A vector network analyzer (Anritsu MS4644B) is used to measure reflections from the probe tips. Dielectric probes are calibrated using open, short, and deionized water standard measurements. Reduced-serum medium is transferred into a clean 50 mm diameter beaker to prepare a sufficiently large and uniform sample of the liquid. The probe tips are submerged a depth of 10 mm into the medium and measured using the vector network analyzer. Complex permittivity information (dielectric constant, loss tangent) is then computed over frequency using the calibration measurements. An empirical model is generated using the permittivity measurements with a previously developed methodology^14^. This model accounts for the frequency-variation of the EM properties of the medium, which significantly improves simulation accuracy.

### Virus and cells

HCoV-229E was obtained from BEI Resources (NR-52726) and propagated as previously described^19^. HCoV-229E stocks were titrated by standard plaquing assay on Huh7 cells^19^. Huh7 cells (JCRB0403) were obtained from the Japanese Collection of Research Bioresources Cell Bank. Cells were cultured in Dulbecco’s minimal essential medium (DMEM) with 10% FBS, 50 U/mL penicillin and 50 $\mu$g/mL streptomycin at 37 $^\circ$C in 5% CO_2_.

### HCoV-229E inactivation assays

HCoV-229E was diluted to 1 x $10^6$ plaque-forming units (PFU)/mL in OptiMEM (ThermoFisher Scientific, 31985062). Aliquots (1 mL) of the diluted virus were distributed into 1.5 mL screw cap tubes (Fisher Scientific 02-681-372) and subjected to the various microwave treatments described. Subsequently, viral infectivity was assessed by plaquing assay. Huh7 cells plated the day before in 12-well plates at a density of 3.5 x $10^5$ cells/well were infected with serially diluted HCoV-229E samples for 2 hours at 37 $^\circ$C. After removing the inoculum, the cell monolayers were overlaid with 1.2% carboxymethylcellulose in DMEM containing 2% FBS and incubated at 33 $^\circ$C in 5% CO_2_ until 4 days post-infection. Cells were fixed and stained with a crystal violet staining solution (1% crystal violet in 17% methanol in H_2_0) to enable visualization of plaques. Plaques were counted to determine the viral titer.

## Data Availability

The datasets used and/or analyzed during the current study are available from the corresponding author on reasonable request.
